# WHO makes new recommendations for Ebola treatments, calls for improved access

**Published:** 2022-09

1.

**19 August 2022** - World Health Organization (WHO) has published its first guideline for Ebola virus disease therapeutics, with new strong recommendations for the use of two monoclonal antibodies. WHO calls on the global community to increase access to these lifesaving medicines.

Ebola is a severe and too often fatal illness caused by the Ebola virus. Previous Ebola outbreaks and responses have shown that early diagnosis and treatment with optimized supportive care —with fluid and electrolyte repletion and treatment of symptoms—significantly improve survival. Now, following a systematic review and meta-analysis of randomized clinical trials of therapeutics for the disease, WHO has made strong recommendations for two monoclonal antibody treatments: mAb114 (Ansuvimab; Ebanga) and REGN-EB3 (Inmazeb).

Developed according to WHO standards and methods for guidelines, and published simultaneously in English and French, the guidelines will support health care providers caring for patients with Ebola, and policymakers involved in outbreak preparedness and response. The clinical trials were conducted during Ebola outbreaks, with the largest trial conducted in the Democratic Republic of the Congo, demonstrating that the highest level of scientific rigour can be applied even during Ebola outbreaks in difficult contexts.

The new guidance complements clinical care guidance that outlines the optimized supportive care Ebola patients should receive, from the relevant tests to administer, to managing pain, nutrition and co-infections, and other approaches that put the patient on the best path to recovery.

“This therapeutic guide is a critical tool to fight Ebola,” said Dr Richard Kojan, co-chair of the Guideline Development Group of experts selected by WHO and President of ALIMA, The Alliance for International Medical Action. “It will help reassure the communities, health care workers and patients, that this life-threatening disease can be treated thanks to effective drugs. From now on, people infected with the Ebola virus will have a greater chance of recovering if they seek care as early as possible. As with other infectious diseases, timeliness is key, and people should not hesitate to consult health workers as quickly as possible to ensure they receive the best care possible.”

The two recommended therapeutics have demonstrated clear benefits and therefore can be used for all patients confirmed positive for Ebola virus disease, including older people, pregnant and breastfeeding women, children and newborns born to mothers with confirmed Ebola within the first seven days after birth. Patients should receive recommended neutralizing monoclonal antibodies as soon as possible after laboratory confirmation of diagnosis.

There is also a recommendation on therapeutics that should not be used to treat patients: these include ZMapp and remdesivir.

All these recommendations only apply to Ebola virus disease caused by Ebola virus (EBOV; Zaire ebolavirus).

“Advances in supportive care and therapeutics over the past decade have revolutionized the treatment of Ebola. Ebola virus disease used to be perceived as a near certain killer. However, that is no longer the case,” said Dr Robert Fowler, University of Toronto, Canada and co-chair of the guideline development group. “Provision of best supportive medical care to patients, combined with monoclonal antibody treatment—MAb114 or REGN-EB3—now leads to recovery for the vast majority of people.”

Access to both these treatments remains challenging, especially in resource-poor areas. These drugs should be where patients need them the most: where there is an active Ebola outbreak, or where the threat of outbreaks is high or very likely. WHO is ready to support countries, manufacturers and partners to improve access to these treatments, and to support national and global efforts to increase affordability of biotherapeutics and their corresponding similar biotherapeutic products, WHO published the first invitation to manufacturers of therapeutics against Ebola virus disease to share their drugs for evaluation by the WHO Prequalification Unit, a crucial step to improve drug access for communities and countries affected by Ebola.

“We have seen incredible advances in both the quality and safety of clinical care during Ebola outbreaks,” said Dr Janet Diaz, lead of the clinical management unit in WHO’s Health Emergencies programme. “Doing the basics well, including early diagnosis, providing optimized supportive care with the evaluation of new therapeutics under clinical trials, has transformed what is possible during Ebola outbreaks. This is what has led to development of a new standard of care for patients. However, timely access to these lifesaving interventions has to be a priority.”

Although WHO was able to make strong recommendations for the use of two therapeutics, there is a need for further research and evaluation of clinical interventions, as many uncertainties remain. Further improvements could be made in supportive care, and in our understanding and characterization of Ebola virus disease and its longer-term consequences, and to ensure continued inclusion of vulnerable populations (pregnant women, newborns, children and older people) in future research.

Available from: https://www.who.int/news/item/22-07-2022-who-releases-global-covid-19-vaccination-strategy-update-to-reach-unprotected

